# Autoimmune polyglandular syndrome type III associated with antineutrophil cytoplasmic autoantibody-mediated crescentic glomerulonephritis: A case report and literature review

**DOI:** 10.1097/MD.0000000000019179

**Published:** 2020-02-14

**Authors:** Shiyuan Tian, Baofeng Xu, Ziwei Liu, Rui Liu

**Affiliations:** a Department of Neurology, China-Japan Union Hospital of Jilin University; b Department of Neurosurgery, First Hospital of Jilin University; c Department of VIP Unit, China-Japan Union Hospital of Jilin University, Changchun, China.

**Keywords:** adult-onset Still disease, antineutrophil cytoplasmic autoantibody, autoimmune polyglandular syndromes, crescentic glomerulonephritis, Hashimoto disease

## Abstract

**Rationale:**

Polyglandular autoimmune syndromes (PAS) are a heterogeneous group of rare diseases characterized by the association of at least 2 organ-specific autoimmune disorders, concerning both the endocrine and nonendocrine organs. Type III is defined as the combination of autoimmune thyroid disease and other autoimmune conditions (other than Addison disease), and is divided into 4 subtypes. We describe a patient with Hashimoto thyroiditis, adult-onset Still disease, alopecia, vasculitis, antineutrophil cytoplasmic antibody (ANCA)-mediated crescentic glomerulonephritis, and hyperparathyroidism. Co-occurrence of these 5 diseases allowed us to diagnose PAS type IIIc. The rare combination of these different diseases has not been reported before.

**Patient concerns:**

A 51-year-old woman was admitted in April, 2019 after the complaint of an enlarged thyroid. She was diagnosed with Hashimoto thyroiditis at the age of 36. At age 40, she was diagnosed with an adult-onset Still disease. Three months before admission, she experienced renal insufficiency. After admission, she was diagnosed with hyperparathyroidism.

**Diagnosis:**

Renal biopsy revealed renal vasculitis and crescentic nephritis. Antineutrophil cytoplasmic autoantibody showed that human perinuclear ANCA and myeloperoxidase ANCA were positive. Therefore, the patient was diagnosed with vasculitis and ANCA-mediated crescentic glomerulonephritis. After admission, parathyroid single-photon emission computed tomography/computed tomography fusion image demonstrated the presence of hyperparathyroidism.

**Interventions:**

The patient was treated with high-dose methylprednisolone pulse therapy (0.1 g/d) for vasculitis and ANCA-mediated crescentic glomerulonephritis, calcium and vitamin D3 (600 mg/d elemental calcium [calcium carbonate] and 2.5 μg/d active vitamin D_3_) for hyperparathyroidism, and levothyroxine sodium (50 ug/d) for Hashimoto thyroiditis.

**Outcomes:**

Up to now, serum thyroid-stimulating hormone, total triiodothyronine, total thyroxine, free triiodothyronine, and free thyroxine were within the normal ranges. Patient's renal function did not deteriorate.

**Lessons:**

We report a patient with Hashimoto thyroiditis, adult-onset Still disease, alopecia, vasculitis, ANCA-mediated crescentic glomerulonephritis, and hyperparathyroidism, which is a very rare combination. We present this case as evidence for the coexistence of several different immune-mediated diseases in the clinical context of a PAS IIIc.

## 1 Introduction

As the incidence of autoimmune disease has gradually increased over the past 10 years, polyglandular autoimmune syndromes (PAS) should be paid significant attention by physicians. PAS are a group of autoimmune disorders characterized by endocrine tissue destruction causing multiple gland malfunction. The classification of PAS proposed in 1980 by Neufeld and Blizzard^[1]^ based on clinical features included 4 main types of PAS: type I, type II, type III, and type IV. In PAS III, autoimmune thyroiditis occurs together with another organ-specific autoimmune disease. PAS III can be further divided into 3 subtypes: PAS IIIa, autoimmune thyroiditis with immune-mediated diabetes mellitus; PAS IIIb, autoimmune thyroiditis with pernicious anaemia; and PAS IIIc, autoimmune thyroiditis with vitiligo, alopecia, and/or other organ-specific autoimmune disease.^[2]^ In this article, we present a rare case of patient affected by PAS IIIc (Hashimoto disease accompanied with vasculitis, antineutrophil cytoplasmic antibody [ANCA]-mediated crescentic glomerulonephritis, adult-onset Still disease, and hyperparathyroidism).

## 2 Case report

A 51-year-old woman was admitted in April, 2019 after the complaint of an enlarged thyroid. Fifteen years before admission, during her annual physical examination, her titers of antithyroid peroxidase (anti-TPO) and anti-thyroglobulin (anti-Tg) increased in the serum. Thyroid ultrasound revealed an enlarged thyroid gland with diffuse hypoechoic lesion. Her free thyroxine (FT4) slightly decreased, and her thyroid-stimulating hormone (TSH) increased. She was diagnosed with Hashimoto thyroiditis and treated with levothyroxine sodium (Na) (50 μg/d). After 3 years, she stopped taking levothyroxine Na. At age 40, she was diagnosed with adult-onset Still disease due to fever, rash, and arthralgia. She was treated with methylprednisolone for 18 days, and her condition sufficiently improved. Hence, she was discharged from the hospital.

**Figure 1:**
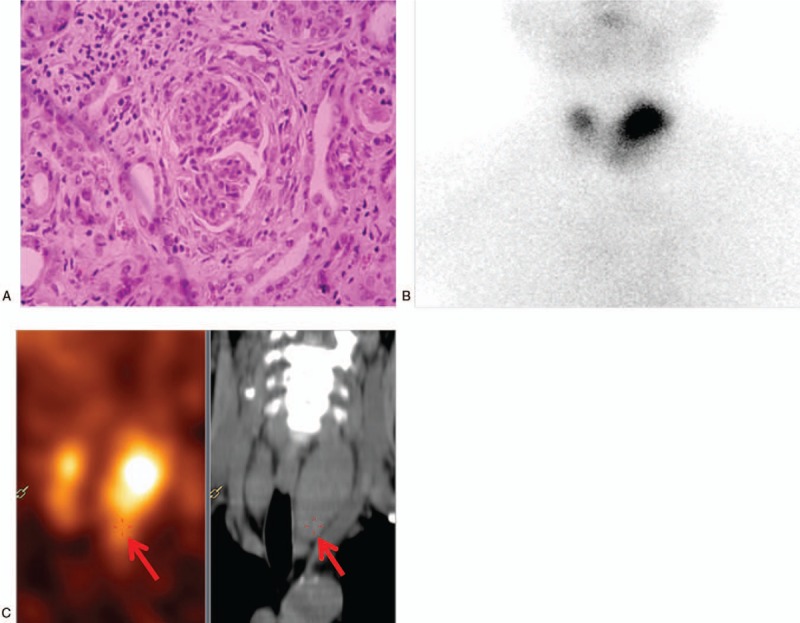
(A) Renal biopsy (hematoxylin and eosin staining ×200) showing the interstitial and perivascular infiltrate comprising lymphocytes and eosinophils, fibrinoid necrosis, and glomerular, parietal epithelial cell hyperplasia. (B) ^99^Technetium scan revealing a high tracer uptake in the left upper thyroid. (C) Parathyroid single-photon emission computed tomography/computed tomography fusion image showing a slightly lower density below the left thyroid with a slightly higher concentration of radioactivity (as indicated by the red arrows).

Three months before admission, she experienced alopecia and renal insufficiency (creatinine 265 μmol/L; glomerular filtration rate 22.03 mL/min). Considering her renal insufficiency, renal biopsy was performed. Light microscopy revealed renal vasculitis and crescentic nephritis (Fig. 1A). Serum antinuclear antibodies were positive (1:100). Antineutrophil cytoplasmic autoantibody showed that perinuclear ANCA and myeloperoxidase ANCA were positive. Therefore, vasculitis and ANCA-mediated crescentic glomerulonephritis were considered. The patient was treated with high-dose methylprednisolone pulse therapy (0.1 g/d).

Upon admission, her body mass index was 21 kg/m^2^, temperature 37.1°C, blood pressure 160/90 mm Hg, and pulse rate 90/min (regular). On physical examination, she presented with diffusely enlarged thyroid. There was slight exophthalmos. Laboratory data on admission were as follows (Table 1): urinalysis showed positive protein (2+), but no glucose, ketonuria, and blood. Blood analysis revealed mild anemia (hemoglobin 86 g/dL). Patient's renal function did not deteriorate. Fasting glucose, serum lipids, and electrolytes were within the normal ranges. The circadian rhythms of serum adrenocorticotropic hormone, cortisol, and renin were normal. Computed tomography scan of the adrenal glands and magnetic resonance imaging scan of the pituitary gland were normal. According to hormone analyses (2019-2-28), serum free triiodothyronine (FT3) (10.76 pmol/L) and FT4 (30.3 pmol/L) levels increased with a suppressed TSH level (0.005 mIU/mL) in the serum. Immunoglobulin G (23.2 g/L) and immunoglobulin M (3.1 g/L) increased. The titers of anti-TPO (600 IU/mL), anti-Tg (4000 IU/mL), and antithyrotropin receptor antibodies (40I U/L) increased. Thyroid ultrasound image showed diffusely enlarged thyroid gland without nodules, confirming the diagnosis of thyrotoxicosis. The radioactive iodine-131 uptake rate showed the following: 2 hours (radioactive iodine uptake rate, 7.16% [reference range 5%–15%]), 4 hours (radioactive iodine uptake rate, 11.34% [reference range 10%–20%]), and 24 hours (radioactive iodine uptake rate, 21.94% [reference range 20%–35%]). We suspected that it was a transient thyrotoxicosis, and the antithyroid therapy (methimazole) was not adapted. The results of thyroid hormone follow-up are shown in Table 2. Additionally, the serum parathyroid hormone (152.4 pg/mL) significantly increased. ^99^Technetium scan demonstrated a high tracer uptake in the left upper thyroid (Fig. 1B), which was associated with thyroid hyperplasia. Parathyroid single-photon emission computed tomography/computed tomography fusion image showed a slightly lower density below the left thyroid with a slightly higher concentration of radioactivity (Fig. 1C). Regarding bone mineral density, an osteoporosis was defined by dual-energy x-ray absorptiometry (the T score of the patient was −3.17 standard deviation [SD] in the lumbar vertebra and −2.63 SD in the right articulatio coxae, lower than the reference value, which was −2.5 SD). Therefore, the patient was diagnosed with hyperparathyroidism and was treated with calcium and vitamin D3 (600 mg/d elemental calcium [calcium carbonate] and 2.5 μg/d active vitamin D3).

**Table 1:**
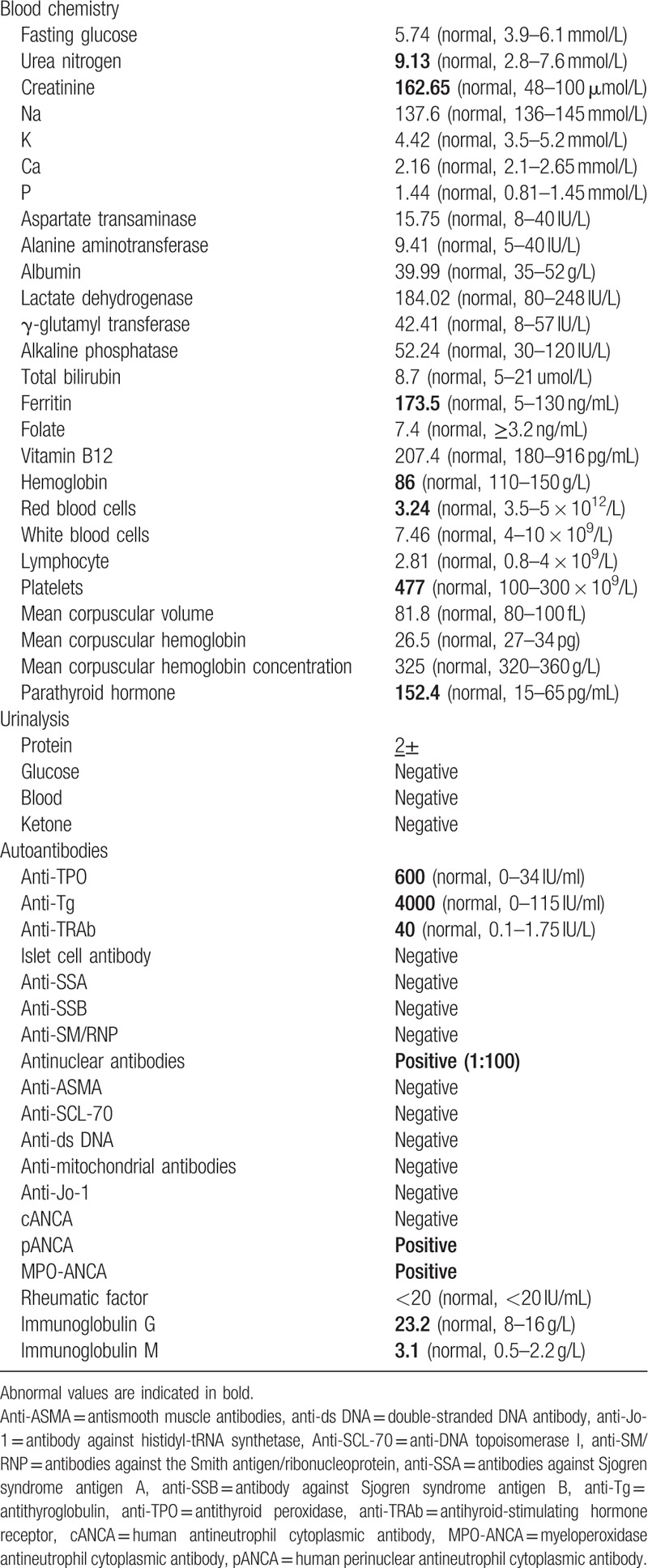
Laboratory data on admission.

**Table 2:**
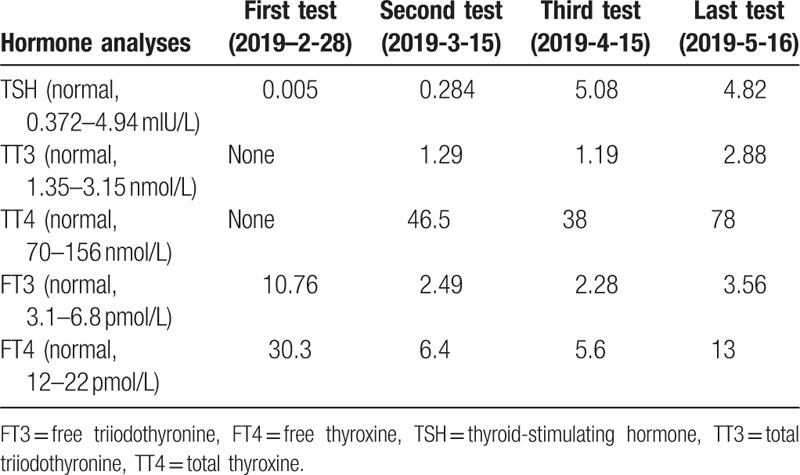
The results of thyroid hormone follow-up.

This study was conducted in accordance with the recommendations of the Ethics Committee of the China-Japan Union Hospital of Jilin University, and all the participants provided written informed consent for the publication of this case report.

## 3 Discussion

Considering the subtle manifestations of Hashimoto thyroiditis and its insufficient clinical features, the early detection of this disease is significantly hard. Hashimoto thyroiditis has a variety of clinical manifestations, which can be characterized by hyperthyroidism, hypothyroidism, and a normal gland. In our case, hormone analyses on admission (2019-2-28) showed increased circulating FT3 (10.76 pmol/L) and FT4 (30.3 pmol/L) with a decreased TSH level (0.005 mIU/mL) in the serum. Hormone analysis after hospital discharge showed that TSH level gradually increased, and FT3, FT4, total triiodothyronine (TT3), and total thyroxine (TT4) gradually decreased. The third hormone analysis (2019-4-15) showed the low level of circulating TT3 and FT3 (TT3, 1.19 nmol/L; FT3, 2.28 pmol/L) and TT4 (TT4, 38 nmol/L; FT4, 5.6 pmol/L) with an increased TSH level (5.08 mIU/mL) in the serum. This was due to the release of thyroxine after thyroid follicle damage, rather than increased thyroxine synthesis; thyroxine levels will decrease over time. Subsequently, hyperthyroidism disappeared and even transitioned into hypothyroidism. In our case, the patient was finally diagnosed with hypothyroidism and received levothyroxine Na (50 μg/d). The last hormone analysis (2019-5-16) showed that the sera TSH, TT3, TT4, FT3, and FT4 were within the normal ranges. In the case of the presented patient, chronic kidney disease was due to hyperparathyroidism. Patients with chronic kidney disease are at risk of calcium and phosphorus metabolism disorders and osteoporosis. The parathyroid gland was stimulated by hypocalcemia and hyperphosphatemia for a long time, and it was easy to secrete a large amount of parathyroid hormone; subsequently, parathyroid hyperplasia was observed.

Polyglandular autoimmune syndrome is defined as multiple endocrine endorgan failure presenting over a variable period of time. Patients with PAS have an increased incidence of autoimmune diseases affecting both the endocrine and nonendocrine organs. The latter disorders include alopecia, vitiligo, pernicious anemia, Addison disease, insulin-dependent type 1 diabetes, rheumatoid arthritis, myasthenia gravis, chronic active hepatitis, and primary biliary cirrhosis. PAS III includes autoimmune thyroid disease plus another autoimmune disorder in the absence of Addison disease. If the other autoimmune disorder is insulin-dependent diabetes mellitus, it is designated as type IIIa. Type IIIb involves pernicious anemia, whereas type IIIc includes vitiligo, alopecia, and/or other organ-specific autoimmune disease. Our patient had Hashimoto thyroiditis, alopecia, adult-onset Still disease, vasculitis, ANCA-mediated crescentic glomerulonephritis, and hyperparathyroidism. Accordingly, she was classified as type IIIc. By reviewing the literature (Table 3  ), we confirm that this is a rare combination that has never been reported. Moss et al^[6]^ described a patient with type IIIc PAS who presented with antibasement membrane antibody disease. They incorporated the antibasement membrane antibody disease into the spectrum of PAS. Shimomura et al^[10]^ reported a case with PAS III associated with Sjögren syndrome and autoimmune neutropenia. They considered autoimmune disorders as the cause of this condition. In our case, multiple autoimmune disorders including autoimmune thyroiditis, adult-onset Still disease, and positive autoantibodies might be associated with the onset of vasculitis and ANCA-mediated crescentic glomerulonephritis. At present, the mechanism of PAS is unclear, but its occurrence is associated with the genetic susceptibility associated with the human leukocyte antigen.^[63]^ Tadmor et al^[64]^ have hypothesized that organs derived from the same embryonal germ layer share common specific antigens. Recent studies have shown that polymorphisms of the T-cell regulatory gene (cytotoxic T-lymphocyte-associated antigen 4) are associated with PAS.^[65]^ Evidently, the immunological mechanisms are crucial in the development of the autoimmune disease, and the intervention of activated self-reacting T cell is considered to be necessary in the majority of the cases to achieve complete destruction of the target organ.^[66]^

**Table 3:**
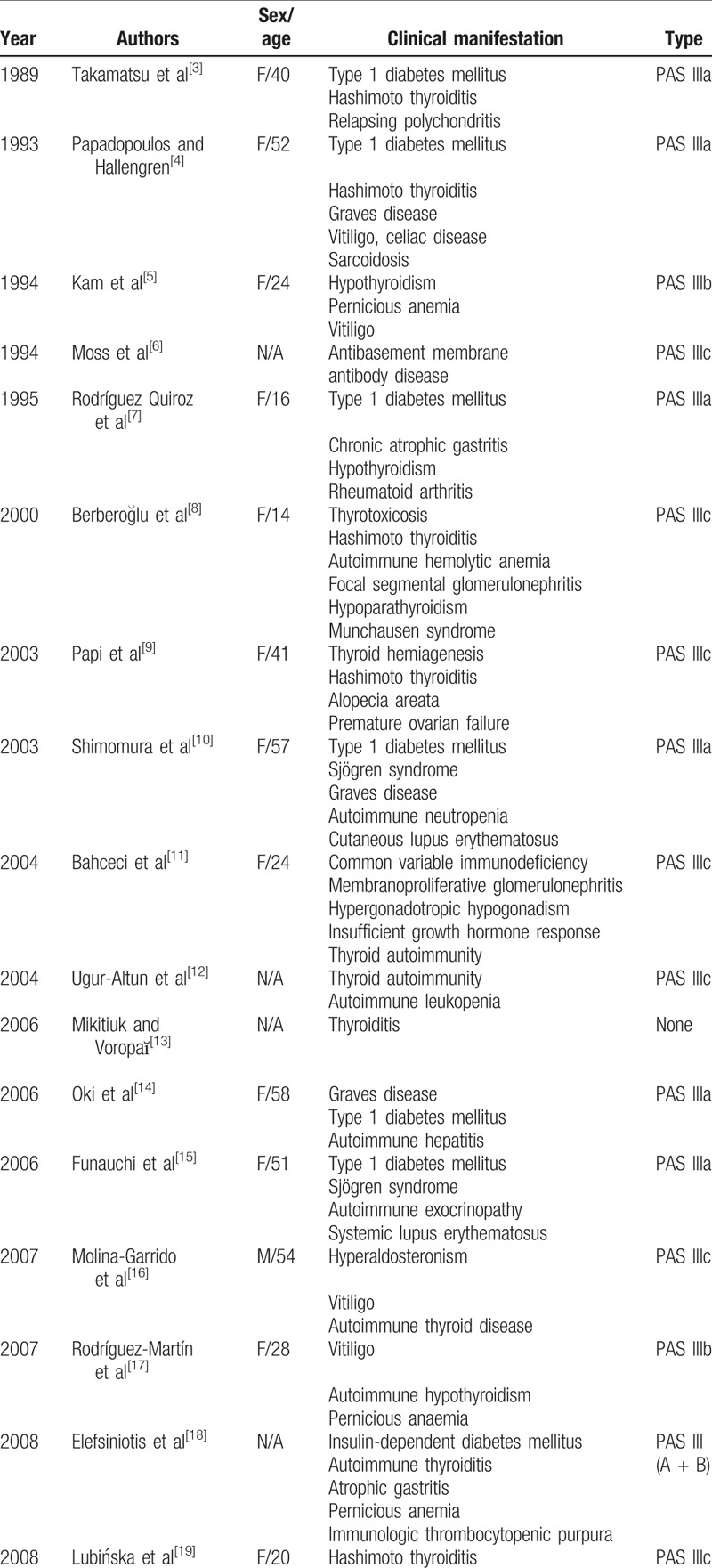
Summary of reported cases with autoimmune polyglandular syndrome type III.

**Table 3 (Continued):**
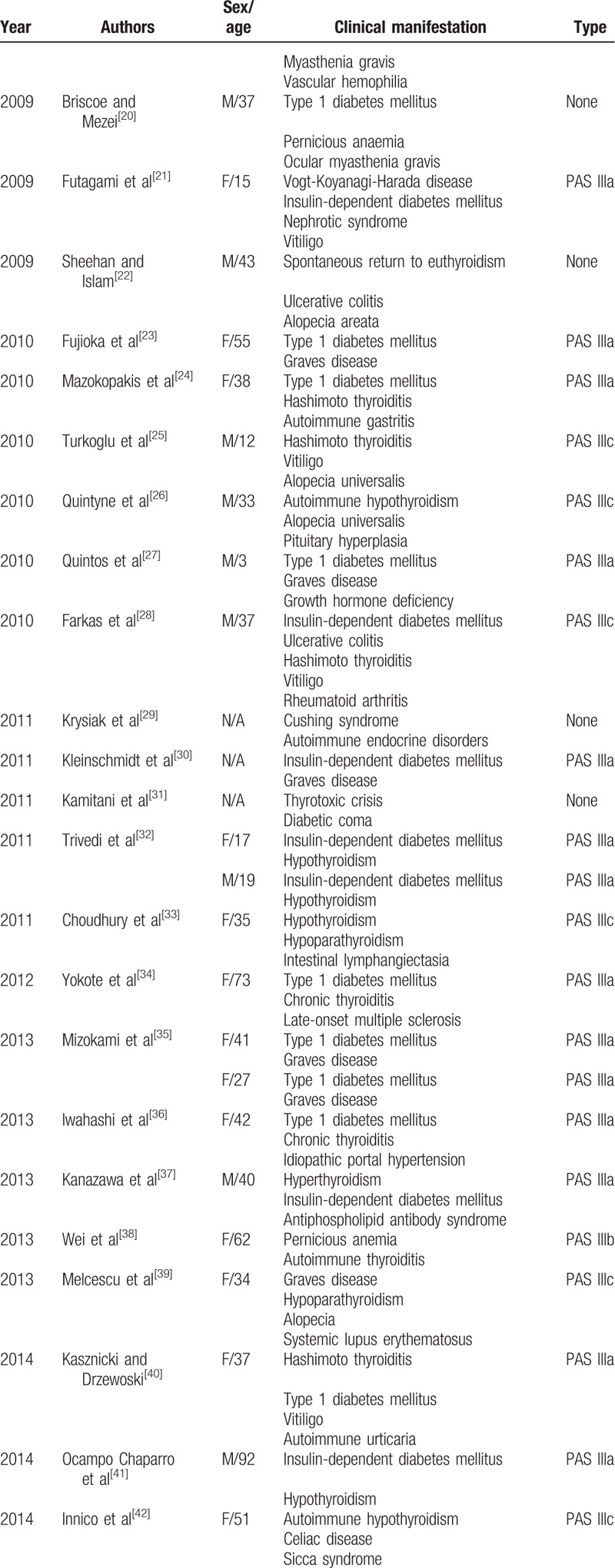
Summary of reported cases with autoimmune polyglandular syndrome type III.

**Table 3 (Continued):**
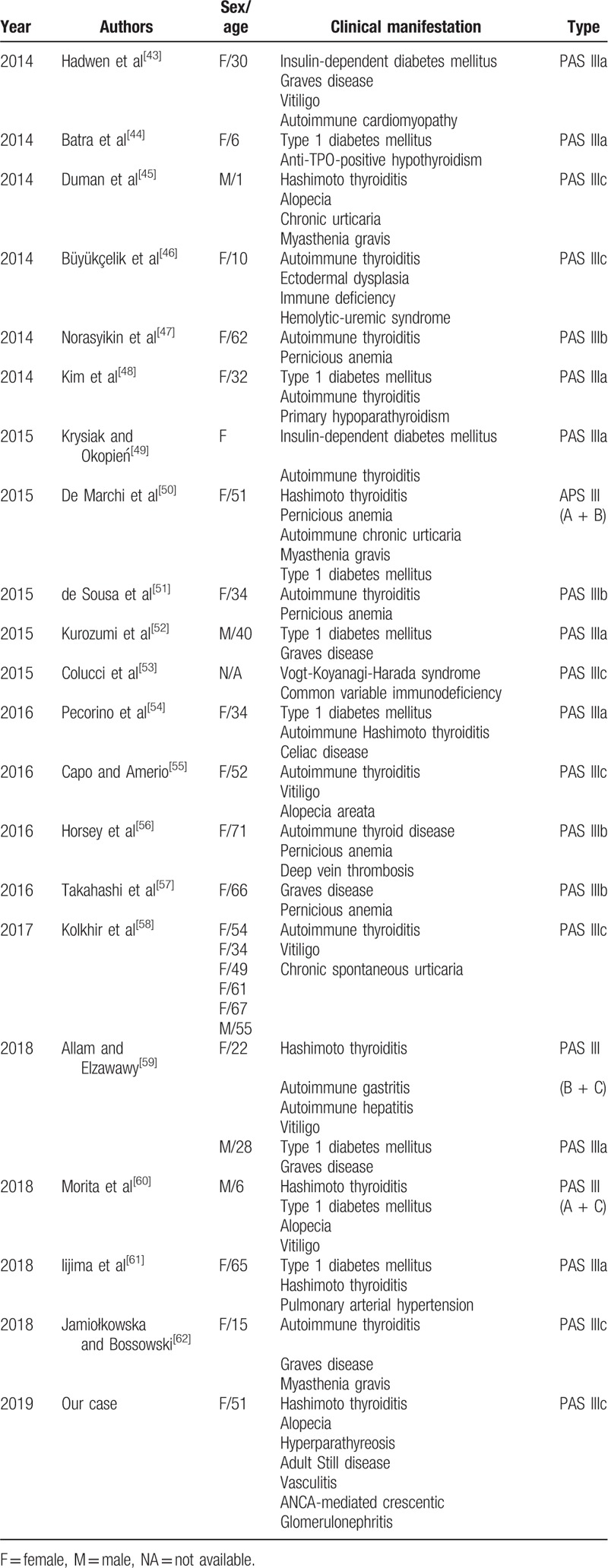
Summary of reported cases with autoimmune polyglandular syndrome type III.

Therapies regarding the different components of PAS III are similar whether they occur as single or in multiple associations with other autoimmune diseases. However, it is worth noting that Hashimoto disease can present as transient thyrotoxicosis; hence, antithyroid drugs and radiotherapy with iodine-131 must be carefully considered when treating Hashimoto disease. Additionally, the thyroid hormone replacement therapy in patients with autoimmune hypothyroidism may result in adrenal failure because thyroxine may enhance hepatic corticosteroid metabolism. Thus, before initiating the therapy with thyroxine, it is crucial to investigate the possible coexistence of an underlying adrenal insufficiency.^[67]^

## 4 Conclusions

We report a patient with Hashimoto thyroiditis, adult-onset Still disease, alopecia, vasculitis, ANCA-mediated crescentic glomerulonephritis, and hyperparathyroidism, which is a very rare combination. We present this case as evidence for the coexistence of several different immune-mediated diseases in the clinical context of a PAS IIIc.

## Author contributions

**Data curation:** Shiyuan Tian.

**Resources:** Zhiwei Liu.

**Supervision:** Baofeng Xu.

**Writing – original draft:** Shiyuan Tian.

**Writing – review & editing:** Rui Liu.
